# Construction of medical scientific data repositories in China: analysis of survey and recommendations

**DOI:** 10.3389/frai.2025.1544200

**Published:** 2025-06-26

**Authors:** Jia Song, Chunqiu Li, Wirapong Chansanam

**Affiliations:** ^1^School of Management, Shenzhen Polytechnic University, Shenzhen, China; ^2^Centre for Modern Industry and SME, Shenzhen, China; ^3^School of Government, Beijing Normal University, Beijing, China; ^4^Department of Information Science, Faculty of Humanities and Social Sciences, Khon Kaen University, Khon Kaen, Thailand

**Keywords:** open access, scientific data, data management, medical open-access repositories, OpenDOAR, re3data

## Abstract

**Background:**

In the context of global open science trends, medical open-access repositories (OARs) promote transparency in research and facilitate the sharing of scientific data. The increase in scientific output necessitates a robust infrastructure to enhance OARs in China.

**Objectives:**

This study aimed to evaluate medical open-access repositories (OARs) in China that are indexed in re3data.org and OpenDOAR.org. The study analyzed data classification, descriptions, retrieval, and the utilization of selected repositories.

**Methods:**

This study ascertained the current status of the Chinese medical OARs by visiting their respective websites and attempted to identify the disciplinary orientation of each OAR. A content analysis approach was utilized to achieve this study’s objective. Twelve Chinese medical open-access repositories were selected from re3data.org and OpenDOAR.org to examine how their information is organized. The data were collected manually from May 1 to 30, 2023, and analyzed using various quantitative techniques to understand the current status of medical scientific repositories in China.

**Results:**

Based on the results, this study proposed the following recommendations: (1) implement multi-dimensional data classification, (2) use persistent data identifiers, (3) formalize the description metadata, (4) enhance advanced retrieval and result set filtering functions, and (5) optimize the preview and interaction features of data repositories.

**Conclusion:**

The scope of this study is restricted to the medical open-access repositories in China as listed on re3data.org and OpenDOAR.org. Therefore, the results of this study are only generalizable within China. The primary focus of research output in China is on medical open-access repositories. This study is essential for assessing China’s current status in research data management within the medical field and its distribution infrastructure in global open science trends.

## 1 Introduction

Scientific data are a vital infrastructure for research, serving as objects, tools, and resources for scientific exploration; effectively managing and enhancing their utilization is essential (1). Medical scientific data are derived from medical research, experiments, clinical services, and health management practices (Guan, 2022); open-access medical scientific data can accelerate the process of scientific research (Liu and Xu, 2014). The International Science Council (ISC) promotes the open sharing of research findings (ISC, 2019). Developing medical big data and promoting new applications are described in the Healthy China 2030 Plan released in 2016 (CPC Central Committee and Council State, 2016).

Open-access repositories (OARs) are vital for freely sharing intellectual knowledge on the Internet and ensuring it is accessible to the public (Sofi et al., 2024). They are considered a new avenue for scholarly communication, allowing researchers to share their work more rapidly with a larger audience (Tmava, 2023). The Directory of Open Access Repositories (OpenDOAR) is the primary directory for searching OARs and their contents (Ibrahim and Beigh, 2019). Since the inception of OpenDOAR in 2005, the number of open-access repositories on the platform has grown significantly, highlighting the influence of the global open-access movement (Sofi and Mir, 2023). The records are reviewed and updated periodically, providing an up-to-date snapshot of the global academic repository landscape (Pinfield et al., 2014). In addition to re3data, this landscape features a rich and diverse collection of data repositories from numerous countries around the world (Khan et al., 2024), aiming to promote the sharing, access, and better visibility of scientific data (Gurikar and Hadagali, 2020). Among the available sources, re3data.org is the most comprehensive registry for searching and identifying data repositories (Gohain, 2021). Medical OARs are essential for promoting data openness (Zuo et al., 2018), providing convenience for adequate access and utilization of medical research data (Ma et al., 2024). Data classification and description serve as the fundamental basis for data services related to medical OARs (Bond and Sheta, 2021). Data retrieval and utilization are integral for promoting OA (open-access) scientific data (Tao and Ye, 2023) and are essential for medical OARs. The standardization of the description of data in China’s national science and technology repositories needs improvement, particularly regarding the granularity and depth of data content description (Si and Wang, 2018). The quality of data retrieval functions and data utilization services in data repositories have attracted the attention of scholars (Wu, 2018). Enhancing the classification, description, retrieval, and utilization of China’s medical scientific data can facilitate better querying, access, and openness of these data. In terms of medical OARs, relevant studies primarily focus on metadata (Archana and Padmakumar, 2023), data organization (Si and Wang, 2018; Wu, 2018), data reuse (Lin et al., 2024), operational status (Bashir et al., 2019), and data sharing (Elvas et al., 2023). However, the use of OARs in medicine and health is a relatively unexplored area of research that requires further investigation (Loan and Sheikh, 2016), and the research on China’s medical OARs is still fragmented and not systematically described. Based on this, the current study aims to advance the research on medical OARs in China.

## 2 Construction of repositories and literature review

### 2.1 Construction of medical OARs

With the support of policy and regulatory frameworks, the development of medical OARs has matured, and there has been progress in managing medical scientific data. The United States and the United Kingdom are the leading countries in the OA movement (Moskovkin et al., 2021) and have prioritized scientific data and formulated relevant policies and laws to regulate the open sharing of medical scientific data (Li et al., 2009). These two countries are primarily involved in developing institutional open-access repositories that form significant components of OpenDOAR (Sofi et al., 2024). In 2011, the UK Research Council issued the “Common Principles on Data Policy,” and this was updated in 2018 (Research Councils UK, 2018). The US National Institutes of Health released the “Data Management and Sharing Policy” in 2003, with an updated version taking effect on 25 January 2023 (NIH, 2020). The UK’s Data Regulation Center utilizes standardized templates designed by DMPonline to create a general medical scientific data management plan, offering personalized health and medical scientific data management services (Donnelly et al., 2010). Since the initial release of the “Protein Sequence and Structure Atlas” bioinformatics database in 1965 (Dayhoff, 1969), databases such as GenBank, EMBL, and DDBJ have emerged (Sindhu and Sindhu, 2023), and repositories for population health data, such as NCBI, CT, and UpToDate, have been established (Fu et al., 2017). The number of scientific data repositories available on re3data.org (Archana and Padmakumar, 2023; Cho, 2019; Sizhu et al., 2018) and OpenDOAR (Maharana and Chakrabarti, 2019; Mir, 2022; Rinehart and Cunningham, 2017) are increasing in number, and the related research continues to expand in various contexts.

China is actively promoting policies and establishing repositories for open access to medical scientific data. In 2018, the “Measures for the Management of Research Data” were implemented (General Office of the State Council, 2018). In May 2019, the State Council issued the “Regulation of the People’s Republic of China on the Administration of Human Genetic Resources” to restrict the sharing of human genetic data; this regulation was revised in 2024 (State Council of the People’s Republic of China, 2024). In June 2019, the Ministry of Science and Technology added the National Center for Genome Sciences Data and other medical repositories to the “List of National Science and Technology Resource Sharing Service Platforms” (Ministry of Science and Technology and Ministry of Finance China, 2019). In April 2021, China implemented the “Biosafety Law,” which aims to establish a comprehensive framework for biosafety management (Standing Committee of the National People’s Congress, 2020). In 2004, construction of the National Center for Population Health Science Data began (Li and Sun, 2015), and in 2015, the BIG Data Center (BIGD) was launched (BIG Data Center (BIGD), 2015). In 2019, the National Microbial Science Data Center (NMDC) was established (NMDC, 2019). Other repositories registered on re3data.org include DEG, GWH, and MiCroKitS, while those registered on OpenDOAR.org include HF-IR, CIB OpenIR, and PSYCH OpenIR.

### 2.2 Review of the related literature

Numerous studies have focused on OARs, and efforts were made to highlight a few studies here, especially from the metadata perspective. Gu et al. (2019) investigated and analyzed the data collection, organization, and analysis processes related to the tranSMART knowledge management repositories. Based on MEDLINE journal selection criteria, Kirkham et al. (2020) identified data organization’s key features and policies in preprint repositories in the biomedical and medical fields. Several studies highlighted the importance of metadata quality and standards for enhancing research data interoperability, including the ways in which different data sources should use appropriate models for storing data and metadata (Kondylakis et al., 2022). The significance of standardized data formats for public resources in the biomedical sector has been highlighted (Swedlow et al., 2021). Removing ambiguity and eliminating duplicate data enhances repository metadata quality (Arencibia et al., 2022). Furthermore, the FAIR (findable, accessible, interoperable, and reusable) principles of scientific data have been implemented in mature natural science fields. A survey was conducted on the global implementation of FAIR principles in research data management (Reisen MVan Stokmans et al., 2019). To support the FAIR Data requirements of the research community, the Chemistry Implementation Network (ChIN) focuses on providing chemical-related data (Coles et al., 2020). Similarly, the Go FAIR Metric Group developed a general indicator framework based on the principle that “one indicator corresponds to only one principle.” This framework provides a template for designing indicators (Azevedo and Dumontier, 2020). The FAIR principles outline requirements, such as findability and interoperability, for data retrieval and metadata description.

Chinese scholars have investigated the current status and experiences of the construction of OARs in the United States, the United Kingdom, Australia, and Canada, e.g., investigating and summarizing the characteristics of government data portal websites in the US and UK regarding scientific data organization, browsing, and retrieval (Li and Xin, 2014). Dengdeng and Feng analyzed the current status of research data storage and sharing repositories in the US, UK, Australia, and Canada from five aspects: policy and legal support, funding sources, construction models, data collection, and data services (Dengdeng and Feng, 2016). Dandan and Jingyuan summarized the experiences of the Canadian Federal Science Data Repository regarding data submission, management, integration, and discovery and offered suggestions for creating a national scientific data repository in China (Dandan and Jingyuan, 2023).

Chinese scholars have also focused on investigating and analyzing the problems in China’s medical OARs, proposing suggestions for improvement, and investigating and analyzing issues related to data description and retrieval across six research data-sharing repositories (Si and Wang, 2018). Jiang et al. (2012) examined the deficiencies in describing and retrieving information from the National Population and Health Science Data Sharing Repositories. Mengxue (2020) compared and analyzed the data description and metadata aspects of multiple domestic and international medical scientific data repositories. Chunqiu et al. (2022) investigated the application of the FAIR principles in China’s medical OARs. Although existing research on China’s open repositories of research data has addressed issues related to data description and retrieval, there remains a lack of comprehensive investigation specifically focused on the classification and utilization of medical scientific data. These studies have provided valuable insights into the practices and lessons learned from constructing scientific data repositories in major developed countries, and their findings can help guide the development of similar repositories and initiatives in China.

Specifically, a survey was conducted on 12 samples of medical OARs in China through online registration and visits to official websites. Based on the survey results, an analysis of the current status and characteristics of data classification, description, retrieval, and utilization of the sample repositories was conducted. This study is expected to promote research by providing suggestions for constructing open medical scientific data repositories.

This study aimed to achieve two primary objectives. First, it sought to classify, describe, retrieve, and utilize data from China’s open scientific data repositories. Second, it explored potential recommendations that could enhance the development of these repositories. This paper aims to provide a current and comprehensive understanding of these repositories. We begin by describing a survey of 12 repositories, analyzing their benefits and conceptualizing their implications. In the concluding sections, we offer suggestions for future successful implementation of open-access data repositories, discuss the limitations of this study, and outline directions for further research.

## 3 Selection of medical OARs in China and survey design

### 3.1 Selection of repositories

#### 3.1.1 Selection approach and principles

**Figure 1:**
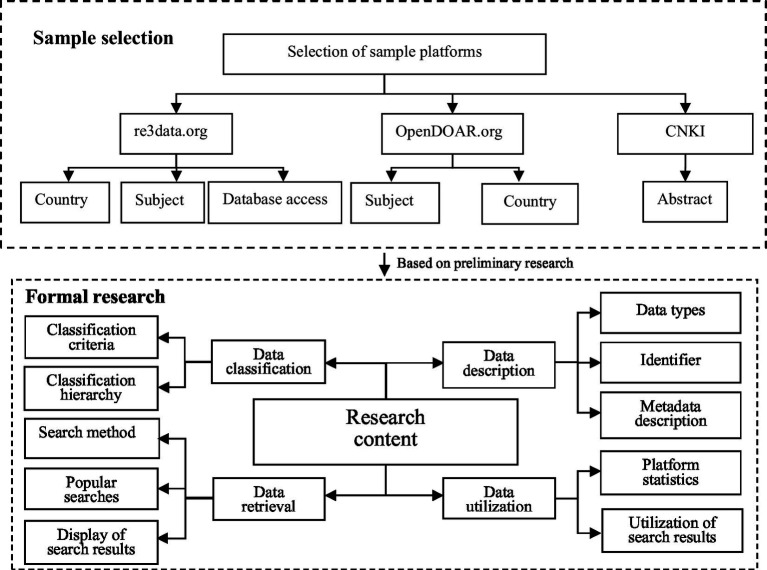
The survey design for this research.

This study focused on medical repositories indexed in re3data.org and Open DOAR, as well as on publications from the Chinese literature extracted from CNKI (China National Knowledge Infrastructure). The proposed methodology for the analysis of twelve different OARs was divided into two parts, as shown in Figure 1. Including preliminary research and formal research, re3data.org is a global directory of research data repositories that provides accessible datasets for researchers, funding agencies, publishers, and academic institutions (Sizhu et al., 2018). China possesses 37 of the largest data repositories, accounting for 24% of all data repositories in Asian countries (Cho, 2019). The search criteria on re3data.org were as follows: “countries = China,” “subject = medicine,” and “database access = open.” Repositories are listed on OpenDOAR.org, which is an authoritative directory of open-access repositories. The search criteria on OpenDOAR.org were as follows: “Subject = Health and Medicine + Biology and Biochemistry,” and “Country = China + Hong Kong + Macao + Taiwan.” Relevant databases of the Chinese literature were searched using the term “title or abstract contains ‘medical scientific data repositories’,” and the results were de-duplicated. The necessary data regarding these repositories were collected manually and entered into a Microsoft Excel file for tabulation. Utilizing multiple sources, this comprehensive approach ensured that the sample encompassed a representative set of medical OARs operated by Chinese institutions or containing Chinese-related data. This provided a solid foundation for the subsequent in-depth investigation.

#### 3.1.2 Selection of sample repositories

After removing duplicate entries and consolidating the repositories from re3data.org and OpenDOAR.org with the relevant literature, we identified a list of twelve accessible open-access medical scientific data repositories for this study. The identified repositories were manually checked. Table 1 provides the respective URLs (uniform resource locator) for these repositories. The types and characteristics of these medical OARs were analyzed for a deeper understanding.

**Table 1:** The research samples of medical OARs in China.

Abbreviation	Name	The primary types of open data	URL
NGDC	National Genomic Data Center	GSA-human, MethBank, Database Commons	https://ngdc.cncb.ac.cn/ (Accessed May 1, 2023)
GWH	Genome Warehouse	Fungi, animals, plants, viruses, etc., and genome data.	https://bigd.big.ac.cn/gwh (Accessed May 1, 2023)
GSA	Genome Sequence Archive	GenBase, GSA Database Commons for human genome, transcriptome, and other omics raw sequences.	https://ngdc.cncb.ac.cn/gsa/ (Accessed May 1, 2023)
MTD	Mammalian Transcriptomic Database	RNA-Seq data for humans, pigs, rats, and mice.	https://ngdc.cncb.ac.cn/mtd/ (Accessed May 1, 2023)
NONCODE	NONCODE	Non-coding RNA data of 17 species, including humans, mice, cows, and rats.	http://www.noncode.org/index.php (Accessed May 1, 2023)
CNGBdb	China National GeneBank Database	Data in the fields of plants, animals, disease, microorganisms, and others.	https://db.cngb.org/ (Accessed May 1, 2023)
NCMI	Population Health Data Archive	Data in the fields of medical basics and research, public health and security, traditional Chinese medicine and pharmacy.	http://www.ncmi.cn/ (Accessed May 1, 2023)
CSTC	China Science and Technology Cloud	Multidisciplinary data, including microbiology, genomics, and basic medicine.	http://www.csdb.cn/ (Accessed May 1, 2023)
BIGD	Science Data Bank	Multidisciplinary data, including biology, clinical medicine, and basic medicine.	https://www.scidb.cn/en (Accessed May 1, 2023)
DEG	Center of BioInformatics	Genomes of bacteria, archaea, and budding yeast.	http://tubic.tju.edu.cn/deg (Accessed May 1, 2023)
MiCroKitS	Midbody, Centrosome, Kinetochore, Telomere and Spindle	Kinetochore, centrosome, midbody, telomere, and spindle data from two fungi and five animals.	https://biocuckoo.cn/joinus.php (Accessed May 1, 2023)
CPLM	Compendium of Protein Lysine Modifications 4.0	105,673 proteins for up to 29 PLM types across 219 species.	https://cplm.biocuckoo.cn/index.php (Accessed May 1, 2023)

### 3.2 Research methods

The methodology used in this study involved extracting metadata elements from the selected repositories on re3data.org and OpenDOAR.org. Additionally, the authors examined the websites of registered medical OARs in China to further explore the research questions. The study’s methodology is illustrated in Figure 1.

To gain a more in-depth understanding of the status of medical OARs, the data were collected from re3data.org and OpenDOAR.org, along with the relevant URLs of medical scientific data repositories, from 1 May to 30 May 2023. The URLs of these repositories were accessed manually to fulfill the research objectives by investigating and analyzing four dimensions: data classification, data description, data retrieval, and data utilization. The extracted data were analyzed using Microsoft Excel. This formed the foundation of the research approach (see Figure 1). Based on the preliminary investigation, the final research content of the study is outlined in Tables 2, 3.

**Table 2:** Survey content regarding data classification and description dimensions.

Dimension	Survey contents	
Data classification	Classification basis	Subject (species, other subjects), source (source unit, source sub-library, other sources), distribution (cell location distribution, chromosome classification of human organs), keywords, data format, scientific data generation method, discipline
	Classification hierarchy	First-level category, second-level category
Data discrimination	Data types	–
	Identifier	Data identifiers, data identifier specifications
	Metadata description	Metadata display format, metadata elements, metadata specifications

**Table 3:** Survey content regarding data retrieval and utilization dimensions.

Dimension	Survey contents	
Data retrieval	Search method	Query components (on-site search engine, query box, drop-down box, checkbox), simple search, advanced search (special entry, Boolean search, faceted search, visual search, field restriction search), data search, fact search, BLAST search
	Popular searches	Popular search terms, hot word clouds, popular search rankings
	Search results display	Display format (scrolling full display, faceted limited display), secondary search function, results and sorting criteria
Data utilization	Platform statistics	Core data statistics, dataset statistics, platform data volume, data source units, chart display
	Utilization of search results	Online file preview, data download, data visualization, data use case analysis, data interaction (external site sharing, favorites, likes)

## 4 Analysis of survey results from sample repositories

The data were analyzed to determine the various characteristics of the retrieved repositories from OpenDOAR and re3data. This section of the article summarizes the findings of the collected data across four aspects: data classification, data description, data retrieval, and data utilization. These findings are displayed in tables.

### 4.1 Current status of repositories concerning the data classification dimension

The data classification dimension was investigated from two aspects: classification basis and classification hierarchy. Table 4 shows the survey results. Table 4 shows the survey results.

**Table 4:** Survey results on data classification dimension.

Survey contents			Sample platform name	Numbers
Classification basis	Theme	Species	GWH, MTD, NONCODE, CNGBdb, NCMI, DEG, MiCroKitS, CPLM	8
		Other topics	NGDC, GWH, NONCODE, CNGBdb, NCMI, CPLM	6
	Source	Source unit	NCMI, CSDB, BIGD	3
		Source sub-library	NGDC, CSDB, DEG, MiCroKitS	4
		Other sources	GWH, NCMI	2
	Distribution	Cell location distribution	MiCroKitS	1
		Chromosome location distribution	MTD	1
		Distribution of human organs	MTD	1
	Keywords		NCMI	1
	Data format		NCMI, CSDB, BIGD	3
	Generation of scientific data		CNGBdb, NCMI	2
	Discipline		NCMI, CSDB, BIGD	3
Classification hierarchy	Level 1		NGDC, GWH, GSA, MTD, NONCODE, CNGBdb, NCMI, CSDB, BIGD, DEG, MiCroKitS, CPLM	12
	Level 2		NGDC, NONCODE, CSDB, DEG, CPLM	5
	Level 3		CNGBdb, NCMI	2
	Level 4		MiCroKitS	1

#### 4.1.1 Integrating characteristics of medical scientific data through multivariate classification

The NCMI comprises various classification bases, including species, data sharing methods, source units, source types, human organ distribution, keywords, data formats, and methods for generating research data. Additionally, eight repositories, including GWH, MTD, and NONCODE, are categorized according to species. MiCroKitS classifies based on the distribution of cell locations, MTD classifies based on the distribution of chromosome locations and human body organs, and NCMI classifies based on the distribution of human body organs. NCMI also provides keyword classification, presenting medical research data corresponding to the top 20 keywords. CNGBdb and NCMI categorize research data based on the method of scientific data generation, which includes observation records, digital processing, instrumental measurements, survey interviews, and simulation analysis. The sample repositories utilize various data classification methods based on the types and characteristics of their open data.

#### 4.1.2 The coexistence of single-level and multi-level classification hierarchies

The research results indicate that among the twelve examined repositories, four OARs (GWH, GSA, MTD, and BIGD) have only one level of classification hierarchy. Out of the total number of OARs, eight have two or more levels of classification. Specifically, MiCroKitS has four classification levels, while CNGBdb and NCMI have three. NGDC, NONCODE, CSDB, DEG, and CPLM have two classification levels. An increased number of detailed classification levels help in identifying and selecting specific data.

### 4.2 Data description dimension

The data classification dimensions are investigated from three aspects: data type, identifier and metadata description. The survey results are shown in Table 5.

**Table 5:** Survey results on data description dimension.

Survey contents			Sample platform name	Numbers
Data types	Scientific and statistical data formats		GSA, MTD, NONCODE, CNGBdb, NCMI, DEG, MiCroKitS, CPLM	8
	Raw data		GWH, GSA, MTD, NONCODE, CNGBdb, NCMI, DEG	7
	Archive data		GWH, GSA, NCMI, DEG, CPLM	5
	Software application		NONCODE, NCMI, MiCroKitS, CPLM	4
	Structured text		MTD, CNGBdb, MiCroKitS	3
	Structured graphics		MTD, NONCODE, CNGBdb	3
	Database		GWH, GSA, CNGBdb	3
	Plain text		DEG, MiCroKitS	2
	Standard office documents		GWH, MTD	2
	Configuration data		MiCroKitS, CPLM	2
	Web-based data		CNGBdb	1
	Picture		NCMI	1
	Audiovisual data		NCMI	1
	Dataset		CSDB, BIGD	2
	Sub-database		NGDC	1
	Others		NGDC, GWH, NONCODE, CNGBdb	4
Identifier	International		CNGBdb, CSDB, BIGD	3
	Domestic general use		NCMI, CSDB, BIGD	3
	Internal customization of the platform		NGDC, GWH, GSA, MTD, NONCODE, DEG, MiCroKitS, CPLM	8
Metadata description	Metadata display format	Web form	NGDC, GWH, MTD, NONCODE, CNGBdb, DEG, MiCroKitS, CPLM	8
		Web page text	NCMI, CSDB	2
		xlsx table	GSA, BIGD	2
	Metadata elements	1–10	CNGBdb, CSDB	2
		11–20	NGDC, GWH, MTD, NONCODE, NCMI, BIGD, DEG, MiCroKitS, CPLM	9
		More than 20	GSA	1
	Follow metadata specifications		NGDC, GWH, GSA	3

#### 4.2.1 Variety of data types and predominantly structured data

The twelve sample repositories include various types of medical scientific data, such as research and statistical formats, raw data, archived data, software applications, structured text, structured graphics, databases, plain text, standard office documents, configuration data, and other data types. Structured data are the primary data types in all twelve repositories except for NGDC, CSDB, and BIGD. The remaining nine sample repositories mainly contain research data, statistical data, raw data, and archived data. NONCODE, NCMI, MiCroKitS, and CPLM include semi-structured software applications, and NCMI includes unstructured graphic and audiovisual data. The medical scientific data repositories can store and recall them through semi-structured and unstructured data.

#### 4.2.2 The adoption of three types of data identifiers

This research found that the sample repositories mainly adopt three types of data identifiers. Firstly, permanent identifiers used for distinguishing unique objects are internationally recognized, such as the Digital Object Identifier (DOI) accepted by CNGBdb, CSDB, and BIGD. Secondly, the unique permanent identifiers commonly used in China, such as NCMI, CSDB, and BIGD, adopt the China Science and Technology Resource (CSTR). Third, internal identifiers for repositories, such as NGDC, GWH, GSA, MTD, and eight other repositories, utilize customized identifiers that are less persistent and compatible compared to the previous two types of identifiers.

#### 4.2.3 Metadata descriptions need to be standardized

This research found that the sample repositories provide metadata through web forms, text on web pages, and XLSX files. Out of the twelve repositories, eight provide metadata in the web form, two present them as web text, and two share them in the XLSX format. The twelve repositories include various metadata elements, with the number of elements ranging from ten to twenty. The NGDC, GWH, and GSA repositories in the sample adhere to the group standard titled “Metadata Standard for Human Genetic Sequencing Raw Data Repertoire” (T/CHIA20-2021), which provides a detailed description of the attributes associated with gene sequencing raw data. The standard was developed based on the data standards set by the International Nucleic Acid Sequence Sharing Consortium and guidelines for database construction. It serves as a valuable reference for the metadata description of data related to health, disease, and other biological projects.

### 4.3 Data retrieval dimension

The data retrieval dimensions are investigated from three aspects: search method, popular searches, and search results display. The survey results are shown in Table 6.

**Table 6:** Survey results on data retrieval dimensions.

Research content			Sample platform name	Numbers
Search method	Query component	Search engine within the site	NGDC, GWH, GSA, MTD, NONCODE, CNGBdb, NCMI, CSDB, BIGD, DEG, MiCroKitS, CPLM	12
		Query box	NGDC, GWH, GSA, NONCODE, CNGBdb, NCMI, CSDB, BIGD, DEG, MiCroKitS, CPLM	11
		Drop-down box	NGDC, GWH, GSA, MTD, NONCODE, CNGBdb, NCMI, CSDB, BIGD, DEG, MiCroKitS, CPLM	12
		Checkbox	MTG, BIGD, DEG, MiCroKitS, CPLM	5
	Simple search		NGDC, GWH, GSA, NONCODE, CNGBdb, NCMI, CSDB, BIGD, DEG, MiCroKitS, CPLM	11
	Advanced search	Special entrance	CNGBdb, NCMI, MiCroKitS, CPLM	4
		Boolean search	MTD, CNGBdb, NCMI, MiCroKitS, CPLM	5
		Faceted search	MTD, CNGBdb, NCMI, CSDB, BIGD	5
		Visual search	MTD, CNGBdb, NCMI, MiCroKitS	4
		Field restriction search	GSA, MiCroKitS	2
	Data retrieval		NGDC, GWH, GSA, MTD, NONCODE, CNGBdb, NCMI, CSDB, BIGD, DEG, MiCroKitS, CPLM	12
	Fact search		CNGBdb, NCMI, CSDB, BIGD	4
	BLAST search		NGDC, GWH, GSA, NONCODE, CNGBdb, DEG, MiCroKitS, CPLM	8
Popular searches	Popular search terms		NGDC, GWH, GSA, CNGBdb, NCMI	5
	Popular word cloud		NCMI	1
	Popular search rankings		NCMI, CSDB, BIGD	3
Search results display	Display form	Scroll to full display	MTD, NONCODE, CNGBdb, MiCroKitS, CPLM	5
		Page-limited display	NGDC, GWH, GSA, NCMI, CSDB, BIGD, DEG	7
	Secondary search function		–	0
	Sort result set by		NGDC, GWH, GSA, NCMI, CSDB, BIGD, DEG, MiCroKitS, CPLM	9

#### 4.3.1 Support for multiple search methods

All twelve sample repositories provide more comprehensive query components, and each sample repository supports a simple search, except for MTD. All twelve repositories feature an on-site search engine and drop-down menus that allow users to refine their search conditions and terms. Additionally, MTD, BIGD, DEG, MiCroKitS, and CPLM include checkboxes to further broaden or narrow the scope of searches based on specific needs. The GSA, MTD, CNGBdb, NCMI, CSDB, BIGD, MiCroKitS, and CPLM sample repositories all support advanced search functions, although their methods vary. CNGBdb and NCMI offer dedicated advanced search portals along with Boolean, faceted, and visualized search options. However, none of these repositories support searches that are restricted to specific fields. In addition, all twelve repositories offer data retrieval capabilities and can be searched to obtain the required information based on user needs. CNGBdb, NCMI, CSDB, and BIGD facilitate factual retrieval and allow for natural language queries, making them user-friendly. Additionally, eight of these repositories feature BLAST (basic local alignment search tool) retrieval, which connects medical scientific data with specialized bioinformatics tools. The sample repositories can be further developed to enhance the advanced search function, improving the accessibility of medical scientific data.

#### 4.3.2 Providing popular search functions

The twelve sample repositories showcase popular searches in various formats. Among them, seven repositories offer a popular search function. The repositories NGDC, GWH, GSA, and CNGBdb provide popular search terms, while NCMI, CSDB, and BIGD present popular search rankings. Additionally, NCMI includes a dynamic word cloud that highlights popular search terms. These top search terms allow users to access the latest and most relevant repository data quickly. They present innovative ideas for data retrieval, while the word cloud visually illustrates the popularity of various repository topics. Trending searches based on high-frequency search terms and research hotspots can help identify cutting-edge trends in the field.

#### 4.3.3 Search results display supports multiple sorting

The twelve sample repositories vary significantly in how they present search results and how they provide support for secondary searches. The repositories can be classified into two display types: scrolling full display and page-limited display. MTD, NONCODE, CNGBdb, MiCroKitS, and CPLM only support a scrolling full display of results. In contrast, seven repositories, including NGDC, support a page-limited display. However, NCMI, BIGD, and DEG do not allow users to select the number of search results displayed on each page; instead, they present a fixed number of results. On the other hand, NGDC, GWH, GSA, and CSDB enable users to choose the number of entries shown on each page, with options for 10, 25, or 50 entries. MTD, NONCODE, and CNGBdb currently do not support sorting of the result set. In contrast, NGDC, GWH, and GSA allow sorting by number, while NGDC, GWH, and GSA also support sorting by description. NCMI, CSDB, and BIGD provide the option to sort by access, and both NCMI and BIGD allow sorting by release time. BIGD also supports sorting by downloads and relevance, while DEG supports sorting by access control functions, gene names, parameter information, and more.

### 4.4 Data utilization dimension

The data utilization dimension is investigated from two aspects: platform statistics and utilization of search results. The search results are shown in Table 7.

**Table 7:** Survey results on data utilization dimension.

Research content			Sample platform name	Numbers
Platform statistics	Core data statistics		NGDC, CPLM	2
	Dataset Statistics		NGDC, GSA, NONCODE, CNGBdb, NCMI, CSDB, BIGD	7
	Total platform data		NGDC, GSA, NONCODE, CNGBdb, NCMI, CSDB, BIGD, DEG, MiCroKitS, CPLM	10
	Data source unit		CNGBdb, CSDB, DEG	3
	Chart display		GWH, GSA, NONCODE, CSDB	4
Utilization of search results	Online file preview		BIGD	1
	Data download		GWH, GSA, MTD, NONCODE, CNGBdb, NCMI, BIGD, DEG, MiCroKitS	9
	Data visualization		GWH, NONCODE, BIGD	3
	Data utilization case study		NCMI	1
	Data interaction	External site sharing	NCMI, CSDB	2
		Favorites	NCMI, BIGD	2
		Like	NCMI, CSDB, BIGD	3

#### 4.4.1 Providing basic statistical functions

Except for MTD, all sample repositories provide statistical data, although the statistics and their presentation are quite basic. Seven of these repositories focus on the number of statistical datasets, while ten are dedicated to the total count of repositories. The NGDC and CPLM repositories track the core data, whereas CNGBdb and DEG monitor the statistical data source units. Additionally, GWH, GSA, NONCODE, and CSDB visually represent the statistical repository data using charts and graphs. Only GWH, NONCODE, and BIGD support the visual presentation of medical scientific data.

#### 4.4.2 Enables users to browse and download search results

All the repositories, except for BIGD, do not support online previewing of files and only present data details for viewing after they have been downloaded. Out of the twelve sample repositories, nine allow data downloads. The NGDC, CSDB, and CPLM do not support data downloads. GWH offers partial data downloads, while CSDB requires an application to access data downloads. The data download formats vary by repository: GWH, MTD, NONCODE, and CNGBdb support downloading GZ files; GSA, NCMI, and BIGD support downloading XLSX files; MTD also supports TXT files; and DEG supports ZIP and DAT files.

#### 4.4.3 Some repositories emphasize user interaction

Data interactions primarily involve actions such as liking, favoriting, and sharing. The CSDB platform allows users to share content through microblogging, while NCMI enables sharing via WeChat, QQ, and microblogging. Both NCMI and BIGD allow users to favorite data and view their favorites. Additionally, NCMI, CSDB, and BIGD support data liking. NCMI also offers tools for analyzing data utilization cases.

## 5 Discussion

OARs provide long-term sustainable storage, preservation, and open access to resources and serve as tangible indicators of an institution’s productivity, thereby increasing the institution’s visibility, prestige, and value (Tmava, 2023). In Asian nations, the open-access movement is expanding (Sofi and Mir, 2023). Data management is a complex issue; many efforts are being made to address privacy, ethical, and intellectual property rights through initiatives such as creative commons and similar activities (Gurikar and Hadagali, 2020). By analyzing the survey results, the analysis framework devised in this research suggests some solutions for selecting OARs. Based on the research results and analysis, China should consider the following recommendations to enhance the openness of medical scientific data and the development of repositories.

### 5.1 Utilization of multi-dimensional data classification

The structure of the twelve medical OARs varies in terms of their specific characteristics and the classification levels of the associated research data. This indicates that medical OARs in China can implement a multi-dimensional data classification system that aligns with the characteristics of the repositories’ data. A detailed classification of medical research data by type and characteristics can enable quicker and more accurate retrieval.

### 5.2 Selection of persistent data identifiers

Out of the twelve sample repositories, eight utilize identifiers defined by the repositories. However, these identifiers lack persistence, stability, and compatibility. Persistent data identifiers are crucial because they enable stable aggregation and effective utilization of datasets. China’s medical OARs can refer to the national mandatory standards for health information and select persistent and stable data identifiers, such as CSTR and DOI, which are widely used both domestically and internationally.

### 5.3 Implementing standardized descriptions for metadata

Currently, the sample repositories have adopted various descriptive specifications for their metadata elements. Only NGDC, GWH, and GSA have selected metadata elements that comply with relevant group standards. The sample repositories can develop appropriate metadata specifications based on published standards in China’s medical field. They can reference both national and international metadata description standards to enhance the integration and utilization of varied medical research data from multiple sources.

### 5.4 Improving the advanced search features and refining the filtering of search results

The sample repositories can enhance the advanced search portal by incorporating various search forms, such as faceted search and visualization search, to improve the user experience when searching for medical scientific data. Among the twelve sample repositories, some lack secondary search functionality, and three do not allow search results to be sorted. Additionally, the logic behind how search results are displayed is unclear, making it difficult to assess and choose relevant results quickly. Therefore, it is advisable for sample repositories to offer a secondary search function and enhance the sorting of result sets, allowing users to rapidly locate desired data based on specific criteria.

### 5.5 Optimizing repository data to improve preview and interaction

The repositories should emphasize their core data, total data volume, data sources, and diverse datasets in visual formats such as charts. Out of the twelve sample repositories, only BIGD provides a preview of data files. NGDC, CSDB, and CPLM do not permit data downloads. It is suggested that these repositories enhance their data preview functionality, provide data download services, and improve interactivity by enabling users to like, collect, and share data on external websites. This will help promote the circulation and utilization of medical scientific data.

This study presents an in-depth analysis of medical scientific data repositories in China, emphasizing their developmental trajectory and areas requiring enhancement. The findings illuminate the interplay between progress and persistent challenges, particularly regarding data classification, description, retrieval, and utilization. The Analysis of Survey Results from the Sample Repositories section reveals noteworthy alignments and critical discrepancies in repository implementation, underlining achievements and opportunities for improvement. The results of the study indicate that current medical scientific data resources primarily focus on genomic phenotypes, while brain imaging datasets, such as the Chinese Color Nest Data Community of Science Data Bank and the National Basic Science Data Center (CCNDC) (Chinese Color Nest Data Community, 2025), have not been fully integrated. Therefore, the study recommends broadening the types of data to include additional categories of medical scientific data, like brain imaging data (Gong and Zuo, 2025), to support more comprehensive research practices.

This research illustrates how China’s repositories are underpinned by robust policy frameworks, such as the “Measures for the Management of Scientific Data” and the “Biosafety Law” (General Office of the State Council, 2018; State Council of the People’s Republic of China, 2024). These initiatives have catalyzed the development of repositories with a focus on open access. However, disparities in metadata standardization, advanced retrieval functions, and user interaction mechanisms persist, as the survey findings demonstrate. While the repositories align with global trends in repository development (Sofi et al., 2024; Gohain, 2021), unique challenges specific to China’s context, such as uneven adherence to metadata standards and limited functionality for advanced searches, hinder their full potential.

From a data classification and description perspective, the literature highlights the importance of adopting multi-dimensional classification and adhering to metadata standards such as the FAIR principles (Rinehart and Cunningham, 2017; Swedlow et al., 2021). Survey results validate these findings, revealing that repositories such as MiCroKitS and CNGBdb utilize advanced multi-level classification systems. However, inconsistencies in metadata adoption still need to be addressed, with only a subset of repositories integrating international standards such as DOI and CSTR (Azevedo and Dumontier, 2020). In data retrieval, the literature emphasizes the necessity of advanced features, including Boolean and faceted searches (Tmava, 2023; Gohain, 2021). Repositories such as CNGBdb and NCMI demonstrate partial implementation of these capabilities, yet gaps in field-specific searches and sorting functionalities suggest room for improvement to align with global benchmarks (Moskovkin et al., 2021). Furthermore, data utilization must be developed, and an increased number of interactive features and visualization tools must be implemented. Although repositories such as BIGD and NCMI exhibit partial adoption of user-friendly interfaces, these features must be more consistent and widespread (Gu et al., 2019).

The growth of repositories in China, as detailed in the Construction of Repositories, reflects the influence of policy-driven initiatives. National frameworks have expedited the establishment of repositories, but their impact varies across institutions, resulting in uneven adoption of advanced functionalities and standardized practices (Ministry of Science and Technology and Ministry of Finance China, 2019). This highlights a critical need for harmonization across repositories to realize the full potential of open-access infrastructure.

The synthesis of findings reveals a dual narrative: China’s repositories are advancing with global trends, yet they must catch up in key areas such as metadata standardization and interactive functionalities. Aligning these repositories with international standards (Chunqiu et al., 2022) and focusing on user-centric improvements will significantly enhance their functionality and accessibility. By leveraging insights from global repositories and addressing the identified gaps, China’s medical data repositories can better facilitate research, improve user engagement, and contribute meaningfully to global scientific collaboration.

In conclusion, this research underscores the imperative to build upon the existing frameworks while addressing the deficiencies that hinder the full realization of the potential of China’s medical data repositories. With a commitment to adopting global best practices and enhancing user-focused functionalities, these repositories can evolve into essential nodes in the international open-access ecosystem, supporting broader scientific innovation and discovery.

## 6 Conclusion

The data from the re3data and OpenDOAR services presented herein provide an important perspective on developing medical OARs in China. The open-access trend in medical scientific data that is described in this article can be explained by data classification, description, retrieval, and utilization. Among these, data description and data retrieval play an important role. In this regard, our study also suggests the construction of medical OARs in China to enhance the management of China’s medical scientific data. Nonetheless, the study results have certain constraints due to limitations in the number of sample repositories, the research duration, and the study’s content. Furthermore, only medical OARs are addressed in this study, and other types of repositories may require different ways of organizing information; an exploration of this matter is not within the scope of this article. Our study also suggests that data-sharing platforms in China need to be more user-friendly. The management of repositories and content can be enhanced for both current and future needs. In the future, China’s medical OARs should enhance data classification, data identifiers, metadata descriptions, and repository functions to promote open sharing and interconnection of research data. Furthermore, topics such as remittance management, the metadata description specifications of medical scientific data, security risk management, and the privacy protection of medical OARs. The researchers’ willingness to share medical science data will also take into consideration. Exploring ways to enhance researchers’ enthusiasm for data sharing is a key factor influencing the level of data openness and the effectiveness of sharing. Establishing a reasonable incentive mechanism for data sharing and clarifying the rights and regulations surrounding data use are essential for researchers to address the concerns and obstacles they encounter during the data-sharing process.

## Data Availability

The original contributions presented in the study are included in the article/supplementary material, further inquiries can be directed to the corresponding authors.
